# Single-atom electron energy loss spectroscopy of light elements

**DOI:** 10.1038/ncomms8943

**Published:** 2015-07-31

**Authors:** Ryosuke Senga, Kazu Suenaga

**Affiliations:** 1 Nano-Materials Research Institute, National Institute of Advanced Industrial Science and Technology (AIST), AIST Central 5, Tsukuba 305-8565, Japan

## Abstract

Light elements such as alkali metal (lithium, sodium) or halogen (fluorine, chlorine) are present in various substances and indeed play significant roles in our life. Although atomic behaviours of these elements are often a key to resolve chemical or biological activities, they are hardly visible in transmission electron microscope because of their smaller scattering power and higher knock-on probability. Here we propose a concept for detecting light atoms encaged in a nanospace by means of electron energy loss spectroscopy using inelastically scattered electrons. In this method, we demonstrate the single-atom detection of lithium, fluorine, sodium and chlorine with near-atomic precision, which is limited by the incident probe size, signal delocalization and atomic movement in nanospace. Moreover, chemical shifts of lithium *K*-edge have been successfully identified with various atomic configurations in one-dimensional lithium compounds.

## Abstract (web summary)

Light atoms are hardly visible through standard microscopy techniques, because of their smaller scattering power and higher knock-on probability. Here, the authors present an approach to probe light atoms by means of electron energy loss spectroscopy, relying on inelastically scattered electrons.

## Graphical abstract

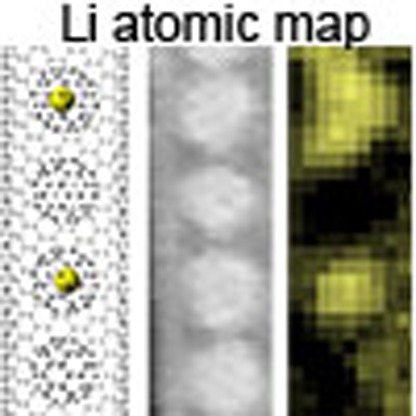

Imaging single atoms of light element by means of transmission electron microscopy (TEM) has two major difficulties compared with heavier elements: the smaller scattering power, which only produces very weak contrast in TEM, and the higher knock-on probability, which allows the atoms to be easily kicked out under the electron beam. In solid state materials with covalent bonds, light elements such as B, C and N (*Z*=5, 6 and 7) have been imaged as single atoms and their chemical assignment by means of electron energy loss spectroscopy (EELS) has been successfully demonstrated^1^,^2^. For those experiments, the materials are relatively resistive to the incident electron beam and the constituent atoms are scarcely kicked out during the observation as long as the acceleration voltage of incident electron beams is kept low.

However this, the lighter element, such as Lithium (Li, *Z*=3), has only been imaged in a three-dimensional crystal as a projection of tens of the identical atoms in a row^3^,^4^ and never been imaged as single atom by means of TEM/scanning TEM (STEM) nor EELS. Even some other light elements such as fluorine (F, *Z*=9), sodium (Na, *Z*=11) or chlorine (Cl, *Z*=17), which are known to be unstable under the electron beam, are also extremely difficult to image as individual atoms, because of their radiation sensitivity^5^. These invisible atoms in TEM often hinder the detailed study of energy storage devices or catalytic particles because one cannot identify the real atomic structures or the local chemical compositions involving those crucial elements.

In this report, we have attempted to capture these light element atoms in EELS chemical map with atomic precision (Fig. 1). EELS is a widely used technique in TEM/STEM for discerning the chemical composition of a sample, and its detection limit has been shown to be sensitive enough to capture a single atom^6^. Also EELS is greatly advantageous for the light element detections because its contrast is not proportional to the atomic number Z but is related to the inelastic cross-section. To capture these light element atom, we have used the ‘peapod' method to locate target atoms in a nanospace, in which a carbon cage such as fullerene or nanotube is used to encapsulate an isolated atom of the specific element^6^,^7^,^8^.

## Results

### Detection of single sodium and chlorine atoms

Figure 2 shows an atomic chain of NaI encapsulated in a double-walled CNT (DWNT) prepared by vapour phase doping. Moving forward from the previous work^7^, we expect that the Na and I ions will align in alternation in the hollow interior of a DWNT (Fig. 2a). A corresponding annular dark field (ADF) image shows only the I atoms (*Z*=53) with sufficient contrast (0.5 nm apart), while the Na atoms (*Z*=11) are apparently missing in between (Fig. 2b). As also confirmed in the quantitative ADF profile in Supplementary Fig. 1, the light Na atoms are invisible here in the ADF image (Supplementary Note 1). Nevertheless, the EELS signal in Fig. 2d does detect the Na *L*-edge at 33 eV, which unequivocally demonstrates the presence of Na atoms. The positions of ‘ghost' Na atoms can then be unambiguously identified in the corresponding EELS chemical map (Fig. 2c).

Single halogen atoms such as F and Cl (*Z*=9 and 17, respectively) are also detectable in a similar way. Figure 3 shows an atomic chain of CsCl in a DWNT. Similar to the previous case, the CsCl atomic chain is also supposed to align in alternation in the DWNT (Fig. 3a). In this case, the heavier Cs atoms (*Z*=55) are clearly visible, while the Cl atoms are apparently missing in the ADF image (Fig. 3b). To confirm the presence of Cl atoms, one must perform an EELS chemical map using the Cl *L*-edge. Figure 3c shows the EELS chemical map of the Cs *M*-edge, which exactly corresponds to the higher contrast in the ADF image, confirming the positions of the Cs atoms. On the other hand, the intensity maxima of the Cl *L*-edge appear between the Cs atoms with hardly visible ADF contrast (Fig. 3d). This demonstrates that even though the Cl atoms are not visible in the ADF image they are indeed captured between two Cs cations in the 1D space of the DWNT. In a similar way, one can also detect much lighter halogen atoms. In Supplementary Fig. 2, we show another example of a CsF atomic chain in which the single F atoms (*Z*=7) can be identified between Cs atoms (Supplementary Note 2).

### Detection of single lithium atoms

Figure 4 shows one of our attempts to capture single Li atoms (*Z*=3). We first encapsulated the Li metallofullerenes inside single-walled CNT (SWNTs) through vapour phase doping. The commercially available [Li^+^@C_60_](PF_6_)^−^, which forms a rock salt type of ionic crystal, was used as the starting material^9^. In this process, a typical ‘Li@C_60_ peapod' (Fig. 4a), in which Li@C_60_ molecules align inside SWNTs, was obtained. Note that PF_6_^−^, which behaves as a counter ion for Li^+^@C_60_ in the crystal, was never observed inside SWNTs unlike other alkali halides. An ADF image of the Li@C_60_ peapod is shown in Fig. 4b. Each C_60_ molecule appears as a round shape in the SWNT. In this ADF image, we can see only the fullerene molecules and SWNT wall, but no contrast corresponding to the Li atoms is found inside the fullerene cage. Since the Li has a smaller atomic number than the carbon, a smaller contrast for Li atoms would be hardly distinguishable in this encapsulating atomic configuration. Moreover, the Li atom is so light that it can easily escape from the electron beam. The EELS chemical map using the Li *K*-edge, however, clearly shows that the presence of Li atoms can be confirmed (Fig. 4c). Among four fullerene molecules, we could detect the Li signals for two of them only. Furthermore, the Li map shows a wider spatial delocalization, which seems to exceed the size of the fullerene cage. In a classical theory, the EELS signal delocalization can be estimated in the range of a few angstroms, but in the case of such a low-lying energy-loss edge (60 eV for the Li *K*-edge), a larger delocalization (up to 0.7 nm) can be reasonably estimated. This is the first direct identification of a Li atom inside a C_60_ molecule and the proof for its endohedral feature^10^.

Alternatively, one can trap the Li atoms in carbon nanotubes (CNTs) as a 1D atomic chain, as with the other alkali halides. Figure 5 shows the 1D LiI crystal grown in a DWNT. This is quite intriguing because, unlike the other alkali halides the 1D LiI crystal does not exhibit the straight atomic chain, but always appears in a zigzag configuration. This could be due to the large difference of the ion radius between Li^+^ (76 pm) and I^−^ (220 pm), or the different interactions expected with the DWNTs^7^ . The thinnest LiI is the 1 × 2 ladder structure shown in Fig. 5a and Supplementary Fig. 3. As seen in Fig. 5b, only the I atoms are visible, and no contrast can be found at the Li positions in the ADF image. On the contrary, the EELS map of Li *K*-edge clearly indicates where the ghost Li atoms are located (Fig. 5c). The zigzag structure in the Li map with a pitch of 0.3 nm is indeed congruent to the structure for I atoms in the ADF image (Fig. 5b).

### Fine structural analysis of Li *K*-edge

The Li *K*-edge fine structure can be derived from these experiments and compared with the other 1D Li structures (Fig. 5e). Interestingly, the Li *K*-edge shows considerable energy shift among these spectra. In the series of 1D LiI with different crystal sizes, the peak positions shifted almost 1.5 eV from the 3 × 3 structure to the 1 × 2 structure. This is probably because the energy to eject the Li 1s electron increases as the coordination number decreases from 4 (surface) to 6 (middle) for the 3 × 3 structure to 3 for the 1 × 2 structure. This is similar to the chemical shift of Li *K*-edge for the bulk structure of Li compounds corresponding to the anionic elements^11^. In the case of Li inside a C_60_ molecule, the peak position (∼63 eV) is much higher than those of 1D LiI crystals (∼60 eV). This fact implies the higher binding energy of Li 1s electrons. The most plausible state could be Li^+^ in which an *L*-shell electron is completely removed from the nucleus by the charge transfer to the outer C_60_ molecule. Such a Li^+^ ion inside a C_60_ molecule seems to be unstable and quite reactive. Indeed, we have also observed the peak position of the Li *K*-edge shifted down to ∼60 eV in the same specimen when two neighbouring C_60_ molecules coalesce (Supplementary Fig. 5). This lower peak position suggests that a Li^+^ inside a C_60_ molecule once forms new bond with C (or even O from atmosphere) and works as a catalyst for the coalescence of two C_60_ molecules, as was observed in transition metal atoms inside fullerene cages^12^. Such a high reactivity also has a good agreement with a recent study for the chemical reaction of Li^+^@C_60_^13^.

## Discussion

The detection of light elements as single atoms demonstrated here was made possible by taking advantage of a ‘cage effect' to overcome the two major difficulties described in the introduction; the smaller scattering power and the higher knock-on probability. In our experiments, the cages composed by CNTs and counter ions (Figs 2, 3 and 5) or C_60_ molecules (Fig. 4) were very effective in preventing escape of atoms by the knock-on effect. In these configurations, the nanotube may protect the cage itself from damages^14^. Without such protections, light atoms are easily kicked out by electron beam even at a low accelerating voltage as shown in Supplementary Fig. 6. In addition, the obtained EELS single-atom contrast, which is related to the inelastic cross-section, is high enough to be isolated from the background noise, while the ADF contrast, which is directly reflected to the scattering power, is too weak to be recognized.

**Figure 1:**
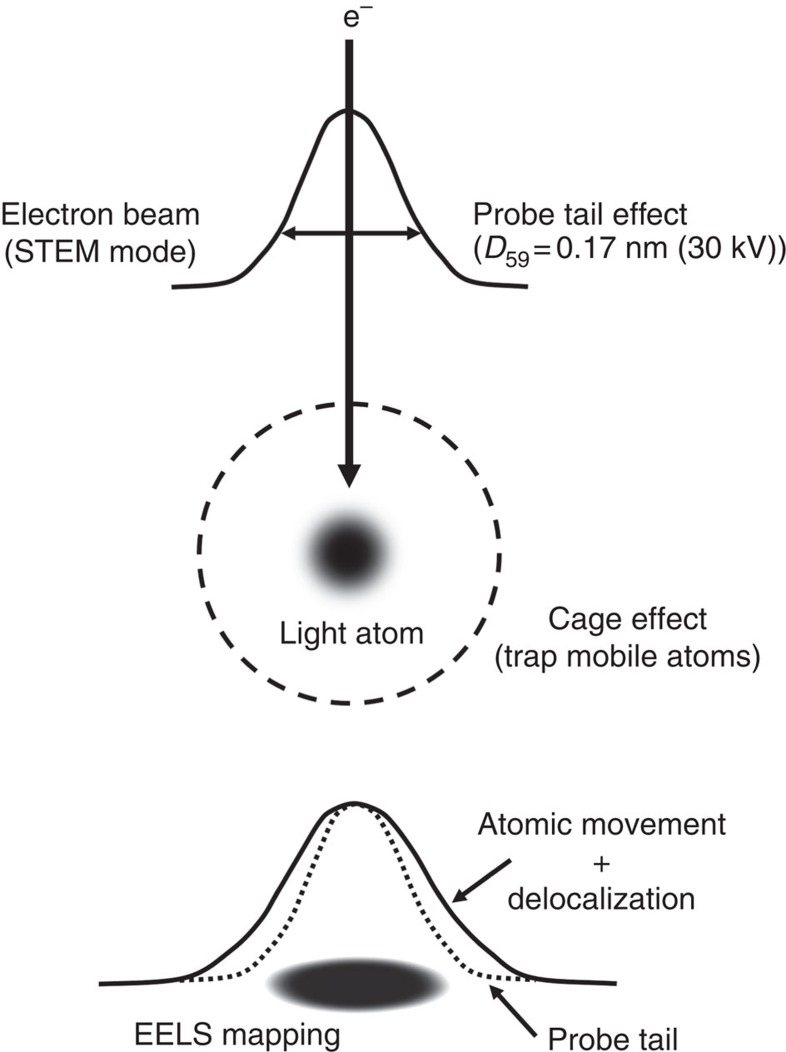
A scheme of EELS chemical map. EELS profile generally involves the electron probe shape, the atomic movement and the signal delocalization. EELS contrast reflects the inelastic cross-section, which is high enough to discriminate a single light atom such as Li. Here we employ the cage effect to trap mobile atoms since the light atom has higher knock-on probability.

**Figure 2:**
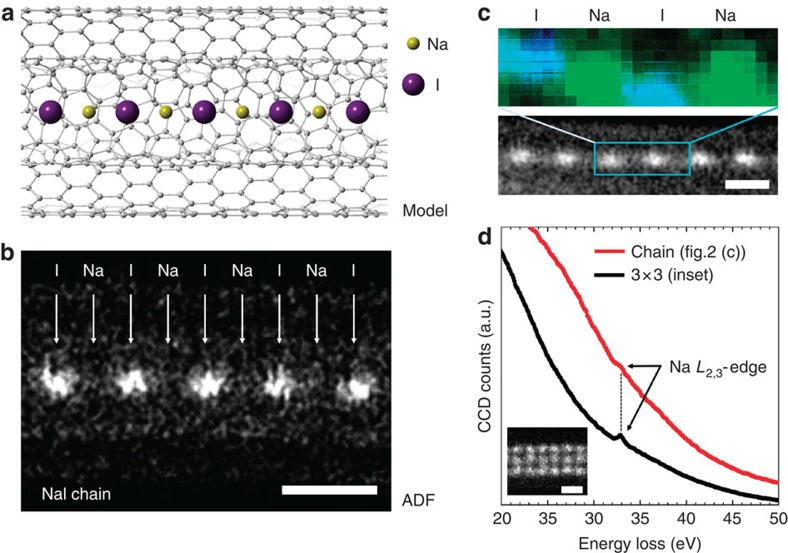
Na atom detection in a 1D NaI crystal. (**a**,**b**) A schematic model and an ADF image of a NaI atomic chain in a DWNT, respectively. In the ADF image, Na atoms are invisible while I atoms present as bright spots. (**c**) An elemental map (upper panel) of a NaI atomic chain shown in a ADF image (bottom panel). (**d**) The EELS spectrum (red line in **d**) taken from the NaI atomic chain shown in **c**. A reference spectrum taken from the 3 × 3 NaI nanowire is also shown (black line in **d**). The Na *L*-edge is clearly visible at 33 eV, even for a single atomic chain of NaI. The EELS chemical map for the Na *L*-edge displays the positions of Na atoms (green spots in the upper panel of **c**) and proves that Na and I are alternatively aligned in the 1D configuration. The Na map is smoothed by a convolution of a 3 × 3 pixel matrix and overlapped with a simultaneously recorded ADF image, which reflects the positions of I atoms (blue spots in the upper panel of **c**). Note that there was a slight specimen drift during the EELS chemical map acquisition. Scale bars, 0.5 nm.

**Figure 3:**
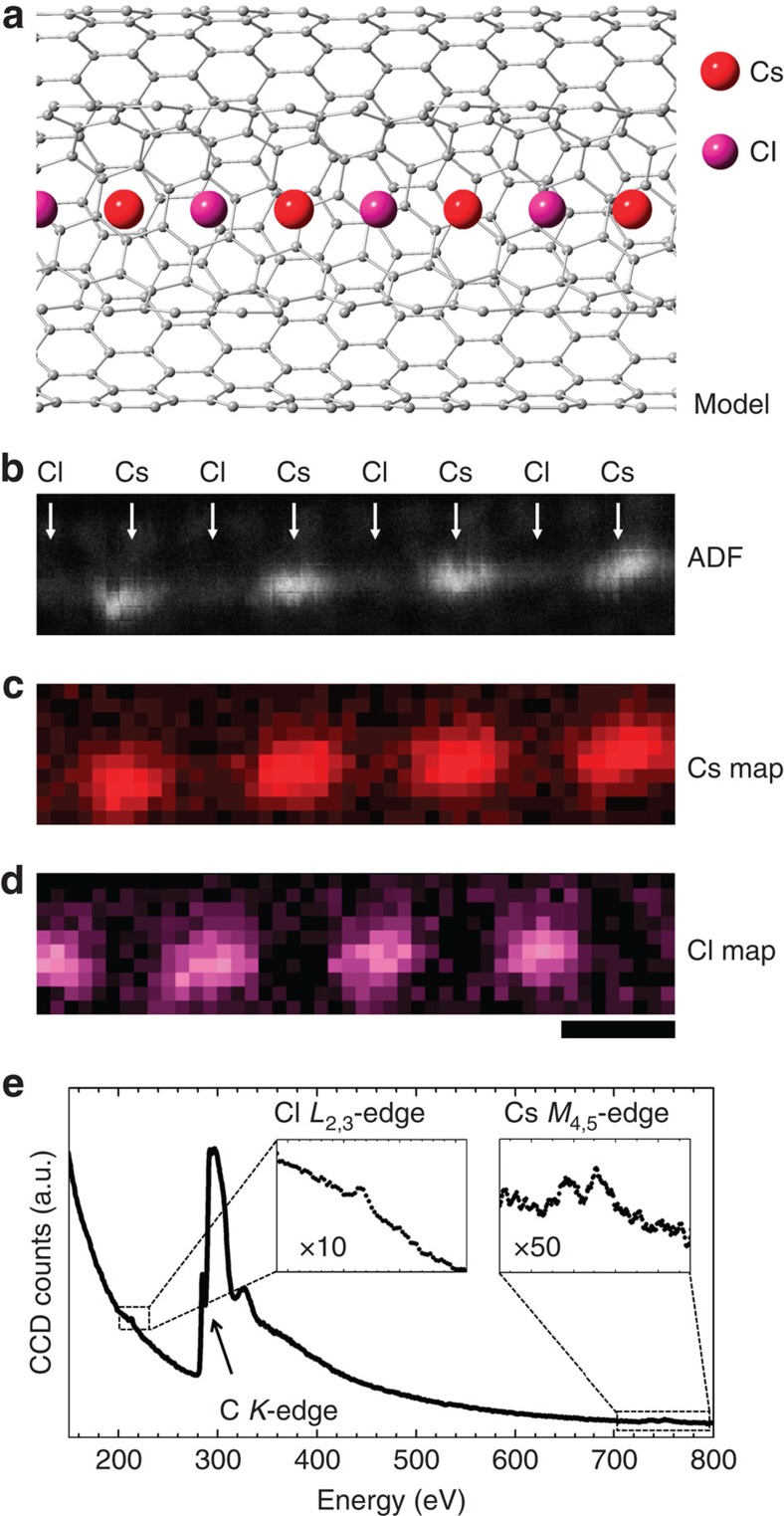
Detection of single Cl atoms. (**a**) Atomic model of a CsCl atomic chain inside a DWNT. (**b**) An ADF image of a CsCl atomic chain. (**c**,**d**) EELS chemical maps for the Cs *M*-edge and Cl *L*-edge corresponding to **b**, respectively. (**e**) An EELS spectrum of the CsCl atomic chain in **b** showing a trace of Cl and Cs, as well as the carbon *K*-edge which corresponds to the DWNT. The ADF image **b** only shows the Cs atomic positions as bright spots which are consistent with the red spots in the EELS chemical map of the Cs *M*-edge **c**. The EELS map for the Cl *L*-edge **d** clearly shows the existence of Cl atoms in between Cs atoms despite of hardly visible ADF contrast in **b**. Scale bar, 0.5 nm.

**Figure 4:**
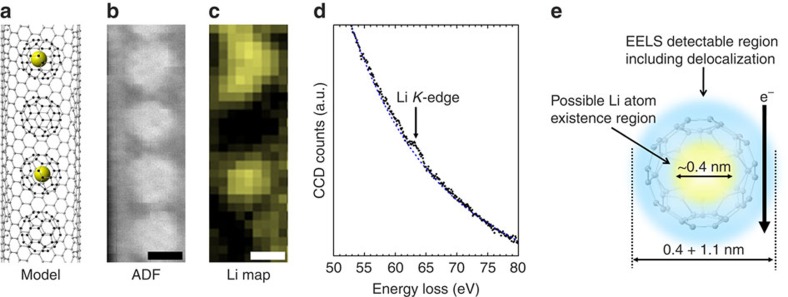
Detection of single Li atoms in a peapod. (**a**) Atomic model of a Li@C_60_ peapod. (**b**) A typical ADF image, which shows fullerene molecules but no visible contrast for the Li atom inside. (**c**) An EELS chemical map of the Li *K*-edge smoothed by a convolution of 3 × 3 pixel matrix. Note that only two of the molecules contain the Li atoms. (**d**) A typical EELS spectrum showing the trace of the Li *K*-edge used for the Li map **c**. (**e**) A schematic model of a Li@C_60_ under the experimental condition. The yellow region in **d** presents a possible mobile space for the Li atom inside the C_60_ molecule (∼0.4 nm), which is roughly estimated from the cage size taking account of van der Waals distance. The EELS detectable region is indicated by the blue circle in **d,** which involves the effect of the atomic movement as well as the delocalization distance of the Li *K*-edge (∼1.1 nm at 30 kV) and is eventually larger than the fullerene cage. Scale bars, 0.5 nm.

**Figure 5:**
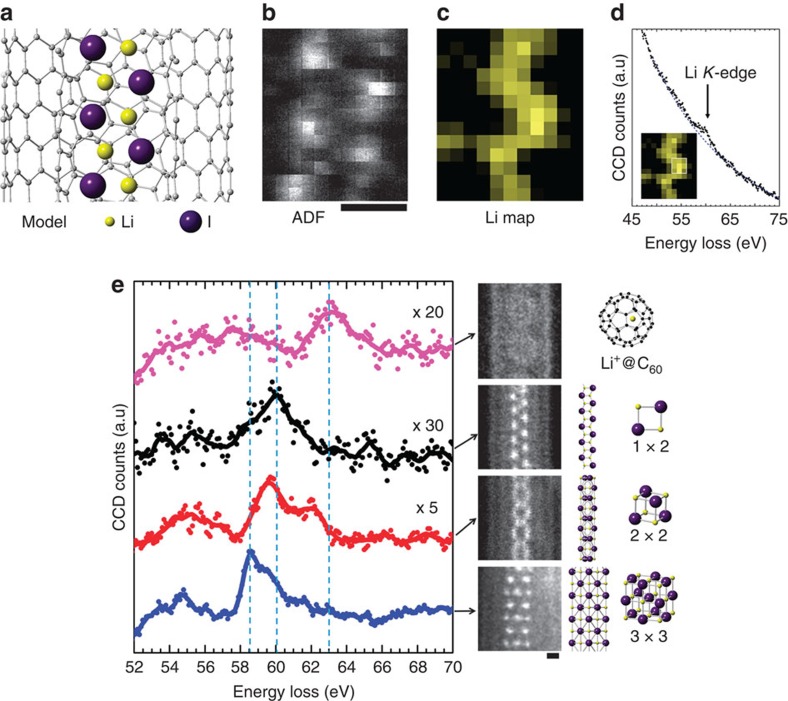
EELS chemical map of a LiI atomic chain. (**a**) Atomic model of the double LiI atomic chain inside a DWNT. (**b**) An ADF image, which only shows the I contrast. (**c**) An EELS chemical map of the Li *K*-edge smoothed by a convolution of 3 × 3 pixel matrix corresponding to **b**. Li atoms are clearly aligned in a zigzag pattern between I atoms displayed as bright spots in **b**. (**d**) An EELS spectrum, confirming the trace of the Li *K*-edge used for the Li map **c**, collected from within a white square in the inset in **d**. The confidence level of the single Li atom detection using Li *K*-edge is sufficiently high (Supplementary Fig. 4 and Supplementary Note 3). (**e**) Fine structure analysis of the Li *K*-edge of various LiI 1D structures. Each spectrum corresponds to the right-side ADF images and models as follows: (from the top) Li@C_60_ inside the SWNT, the 1 × 2 structure, the 2 × 2 structure and the 3 × 3 structure of LiI, respectively. There is a systematic variation for the Li *K*-edge with those atomic configurations. The peaks apparently shift to the higher energy as the coordination number decreases for Li from 4 to 6 (the 3 × 3 structure) down to 2 (the 1 × 2 structure) or 0 (the Li@C_60_). Scale bars, 0.3 nm.

Along with the above discussion, the effects of electron probe tail also should be taken into account because the probe size (that is, *D*_59_=0.17 nm in which the 59% of the beam current is involved at 30kV) cannot be negligible in this scale. Therefore, EELS profile recorded across a single atom is supposed to reflect the following three factors—the effect of an electron probe tail, the atomic movement in a nanospace and the EELS delocalization (Fig. 1). Table 1 summarizes the full width at half maximum (FWHM) of our experimentally obtained EELS profiles recorded across a single atom of various elements as EELS detectable distance, as well as parameters for the three factors. The probe size was carefully evaluated considering the experimental condtions^15^,^16^. The size of nanospace in Table 1 is estimated by the width of the empty space surrounded by nearest neighbour atoms. In reality, since atoms cannot get closer beyond the certain distance because of coulomb repulsion force; the mobile area should be smaller than the value. The theoretical delocalization distance in Table 1 is estimated based on a classical theory^17^ (Supplementary Note 4). This value is basically inversely proportional to the absorption energy and becomes larger in low loss edges such as Na *L*-edge (∼33 eV) or Li *K*-edge (∼60 kV).

Among these three factors, the effects of electron probe tail and the atomic movement must be also involved in the ADF profiles simultaneously recorded and therefore the EELS delocalization effect can be distinguished by a direct comparison between ADF and EELS profiles for heavier atoms (Supplementary Fig. 7). For example, the EELS profile of a Cs atom in a CsCl atomic chain is 0.1 nm larger than its ADF profile in FWHM. The difference can be interpreted as the EELS delocalization effect and indeed comparable to the theoretical value listed in Table 1. Although the degree of EELS delocalization for light elements cannot be directly estimated in the same manner because there is no contrast detectable in their ADF images to compare, the larger EELS detectable distance (0.3–0.7 nm) measured for the Li atom inside a C_60_ molecule should reflect mostly the EELS delocalization as well as the atomic motion, which is, however, limited within the fullerene cage (0.7 nm).

Moreover, we show that the chemical states of light elements can be unambiguously assigned at an atomic scale. Indeed, we found the systematic variations both in the Li and F *K*-edge with different atomic configurations showing increasing coordination number. Such chemical state information will be the key to know the mechanism and the atomic procedure of existing fundamental chemical reactions. For example, the precise identification of charge state of Li atoms at each reaction stage in ion batteries would be of great importance in understating their performance. Therefore, our method, which enables to assign those light atoms with their detail chemical information at a single atomic scale, is of great consequence from the viewpoints of fundamental science and industrial applications. Our method presented here is still on the premise of the existence of the ‘cage', which prevents the escape of light atoms. However, we believe that further precise studies and controls of the factors dictating the detection limit of light atoms with EELS discussed above will open new concepts for the characterizations of ‘cage free' materials or radiation-sensitive matters such as biomolecules.

## Methods

### STEM and EELS observations

For STEM and EELS, we used a JEM-2100F equipped with a delta corrector and cold-field emission gun. The acceleration voltage was set to 60 and 30 kV to reduce damage to the samples by electron beams. The probe size is ∼0.1 nm in FWHM for both 30 and 60 kV. The diameters of *D*_59_ and *D*_90_ in which 59% and 90% of the beam current are involved are estimated as 0.17 nm and 0.51 nm at 30 kV, respectively^16^. For EELS analysis, we used a GIF Quantum spectrometer (Gatan) specialized for low-voltage operation. The convergence and EELS collection semiangles were 48 (40 mrad) and 59 (63 mrad), respectively, at 60 (30 keV). Typical electron-beam currents during the experiments were a∼10–20 pA. The EELS spectrometer was set to 0.1–0.5 eV per channel dispersion. The exposure time for 1 pixel was 0.1–0.2 s. The degree of delocalization in EELS under this condition is presented in Supplementary Fig. 8.

### Sample preparation

We used ionic crystals (NaI, CsF, CsCl and LiI) in which light and heavy atoms are paired. They were encapsulated in CNTs by heating them at 400–500 °C in glass or quartz tubes fused in vacuum. Their crystal size was determined by the diameter of the CNTs. NaI, CsF and CsCl atomic chains (Supplementary Fig. 1), and LiI double-atomic chains (Supplementary Fig. 3) were formed inside DWNTs with inner diameters of <1 nm, prepared using a high-temperature pulsed arc discharge^18^. In this process, we found an interesting atom exchange. If LiI was heated along with DWNTs in a commercial glass tube, the resultant material inside the DWNTs always consisted of NaI atomic chains. This happened because the Li ions reacted with the glass tube and were replaced by Na ions contained therein.

## Additional information

**How to cite this article:** Senga R. & Suenaga K. Single-atom electron energy loss spectroscopy of light elements. *Nat. Commun.* 6:7943 doi: 10.1038/ncomms8943 (2015).

## Supplementary Material

Supplementary Information Supplementary Figures 1-8, Supplementary Note 1-5 and Supplementary References

## Figures and Tables

**Table 1:** EELS detectable distance for single atoms and considerable factors involved in the experimental values.

Element (*Z*)	Structure	Measured edge	EELS detectable distance (nm)	Probe size *D*_*59*_(nm)	Size of nano space (nm)	Theoretical EELS delocalization *L*_*50*_ (nm)
Li (3)	Li@C_60_ (Fig. 4)	∼63 eV *K*	0.53 (±0.18)	0.17	0.71	0.53
Li (3)	LiI 2 × 1 (Fig. 5)	∼60 eV *K*	0.14 (±0.07)	0.13	0.43	0.66
F (9)	CsF chain (Supplementary Fig. 2)	∼690 eV *K*	0.13 (±0.08)	0.13	0.49	0.11
Na (11)	NaI chain (Fig. 2)	∼33 eV *L*_2,3_	0.13 (±0.04)	0.13	0.54	1.03
Cl (17)	CsCl chain (Fig. 3)	∼200 eV *L*_2,3_	0.17 (±0.06)	0.13	0.62	0.27
I (53)	CsI chain (ref. 12)	∼660 eV *M*_4,5_	0.20 (±0.10)	0.13	0.68	0.11
Cs (55)	CsI chain (ref. 12)	∼730 eV *M*_4,5_	0.19 (±0.10)	0.13	0.68	0.10

EELS , electron energy loss spectroscopy; FWHM, full width at half maximum.

‘EELS detectable distance' is defined as the average length for the FWHM of each peak in the chemical maps of our results. Interestingly, some of these values are smaller than the theoretical EELS delocalization *L*_50_ (Supplementary Note 5). ‘Probe size *D*_59_' is defined as the diameter that contains 59% of the total probe current was estimated in refs 15, 16. ‘Size of nanospace' indicates the width of the space surrounded by nearest neighbour atoms. ‘EELS delocalization *L*_50_' in which 50% of the inelastically scattered electrons are contained is estimated by equation 3 in Supplementary Note 4. Only the values for Li@C_60_ are estimated at 30 kV of accelerating voltage. Others are estimated at 60 kV.
