# Supplementary material for: Single-atom electron energy loss spectroscopy of light elements
Source: Nat Commun. 2015 Jul 31;6:7943. doi: 10.1038/ncomms8943 (PMC4532884; doi:10.1038/ncomms8943)
Supplement: Supplementary Information — Supplementary Figures 1-8, Supplementary Note 1-5 and Supplementary References [file ncomms8943-s1.pdf]

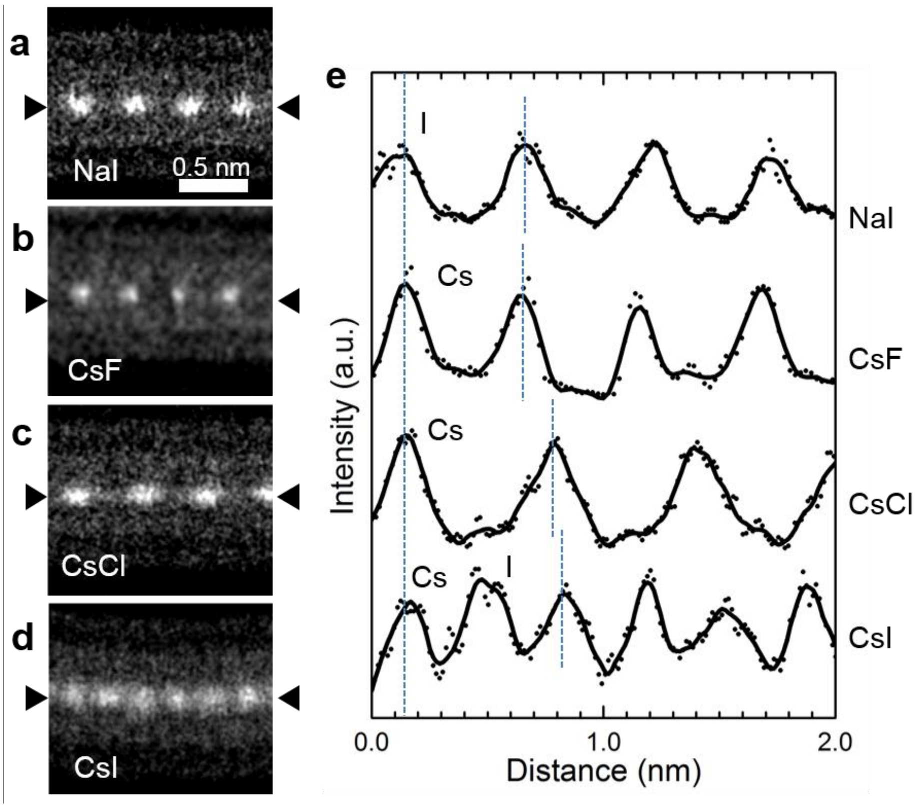

Supplementary Figure 1: ADF images and profiles for several types of atomic chains encapsulated in DWNTs. (**a**–**d**) ADF images of NaI, CsF, CsCl, and CsI atomic chains encapsulated in DWNTs, respectively. (**e**) Comparison of the ADF profiles along the chains.

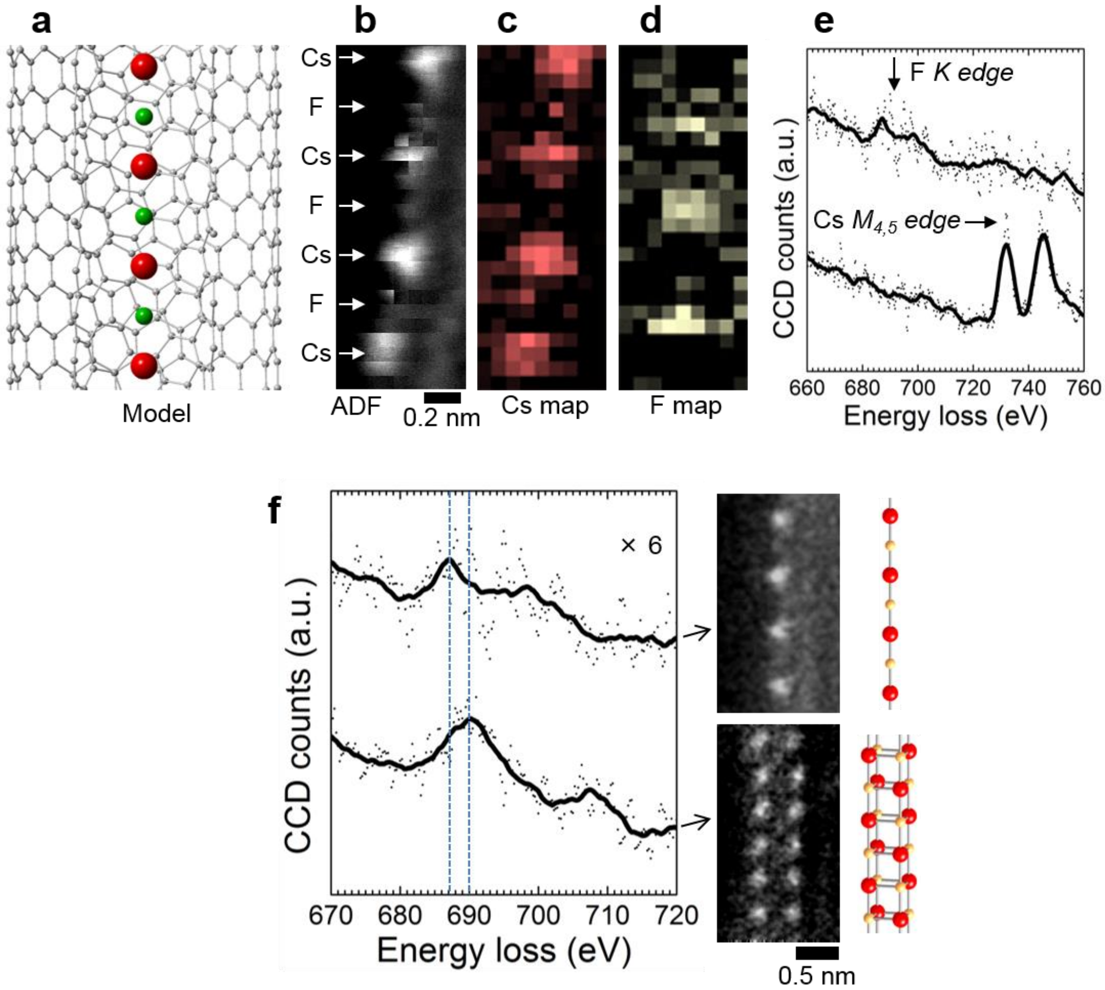

Supplementary Figure 2: Detection of single fluorine atoms. (**a**) The atomic model of CsF atomic chain inside DWNT. (**b**) An ADF image which only shows the Cs atomic positions. (**c**) EELS chemical map of the Cs *M*-edge. (**d**) EELS chemical map of the F *K*-edge. (**e**) A typical EEL spectrum showing a trace of the F *K*-edge (upper) and Cs *M*-edge (bottom). (**f**) Comparison of the F *K*-edge for the atomic chain (upper) and 2 × 2 structure of CsF (bottom).

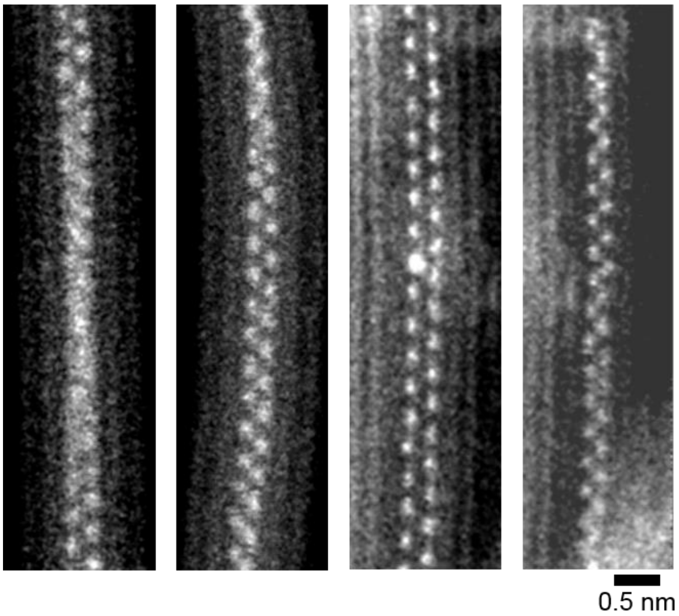

Supplementary Figure 3: ADF images of the 1 × 2 ladder structure of LiI.

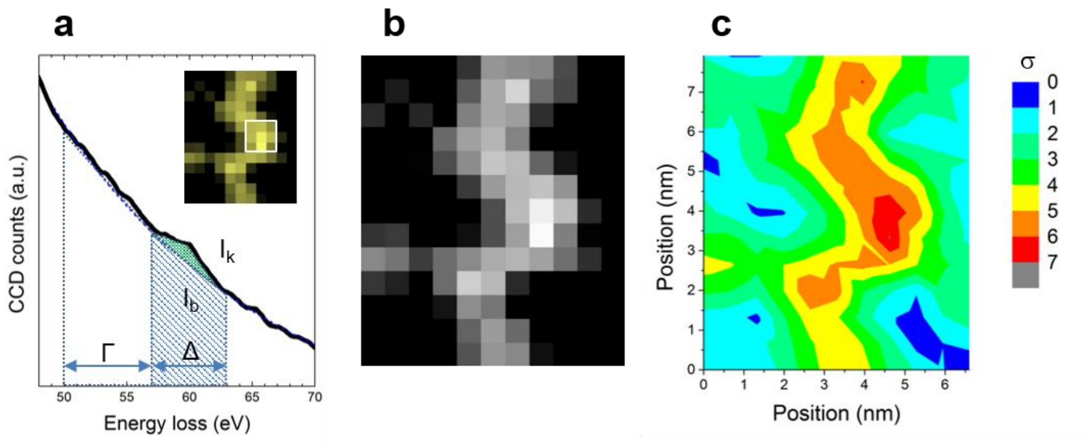

Supplementary Figure 4: Noise level of the Li signal. (**a**) EEL spectrum of the Li *K*-edge gathered from a 3×3 pixel matrix (i.e. 0.2 nm × 0.2 nm) indicated by the white square in the inset. (**b**) EELS Li map. (**c**) Color map corresponding to the standard variation of the signal noise σ.

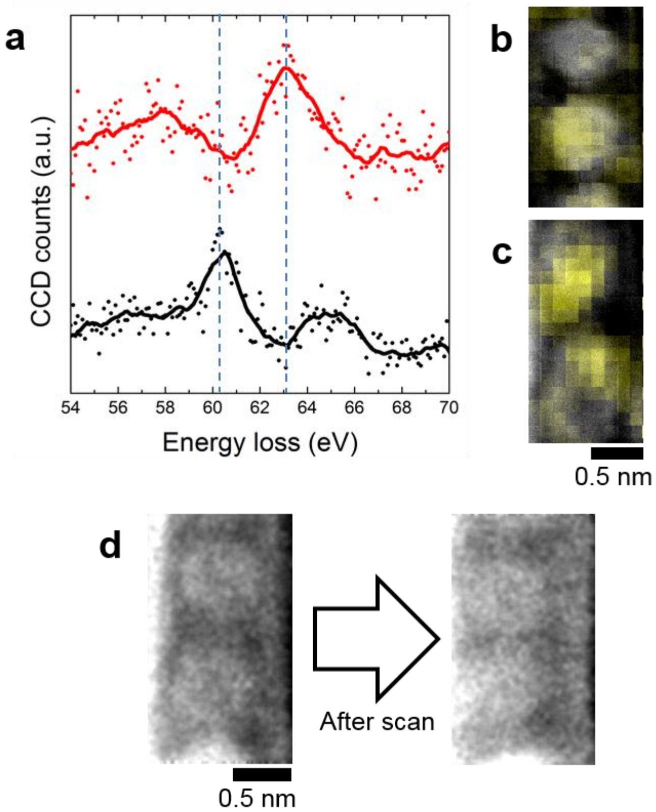

Supplementary Figure 5: Variation in Li *K*-edge corresponding to the chemical state of Li atoms inside C_{60} molecules. (**a**) Two different types of Li *K*-edge corrected for the Li atoms inside C_{60} molecules. (**b**, **c**) Li maps stacked on ADF images corresponding to the spectra indicated by the red and black dots in **a**, respectively. (**d**) ADF images before and after the EELS measurement for the spectrum indicated by black dots in **a**. After the measurement, two C_{60} molecules started to fuse.

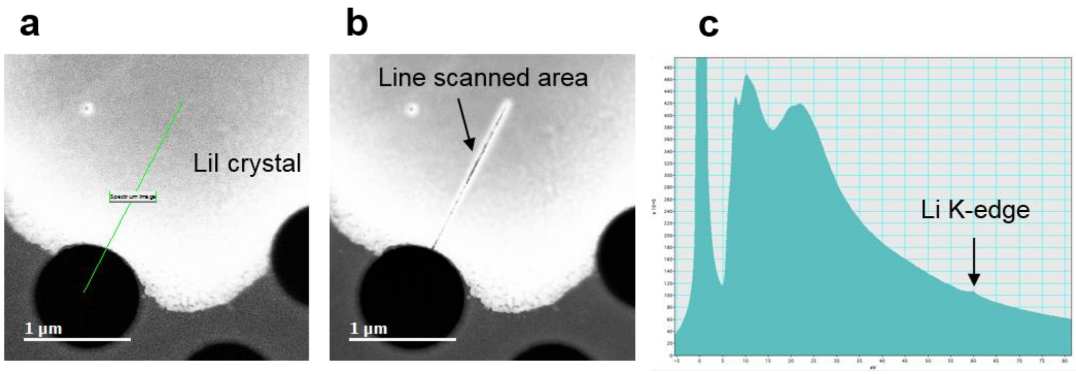

Supplementary Figure 6: Beam damage on a LiI bulk crystal at 60kV. (**a**,**b**) Low magnification STEM images of a LiI bulk crystal deposited on a microgrid before and after an EELS line scan on the crystal along the green line in **a**, respectively. (**c**) A sum of EEL spectra corrected during the line scan. The Li K-edge intensity quickly decreases as the duel time. The scanned area was completely damaged by the electron beam as shown in **b**.

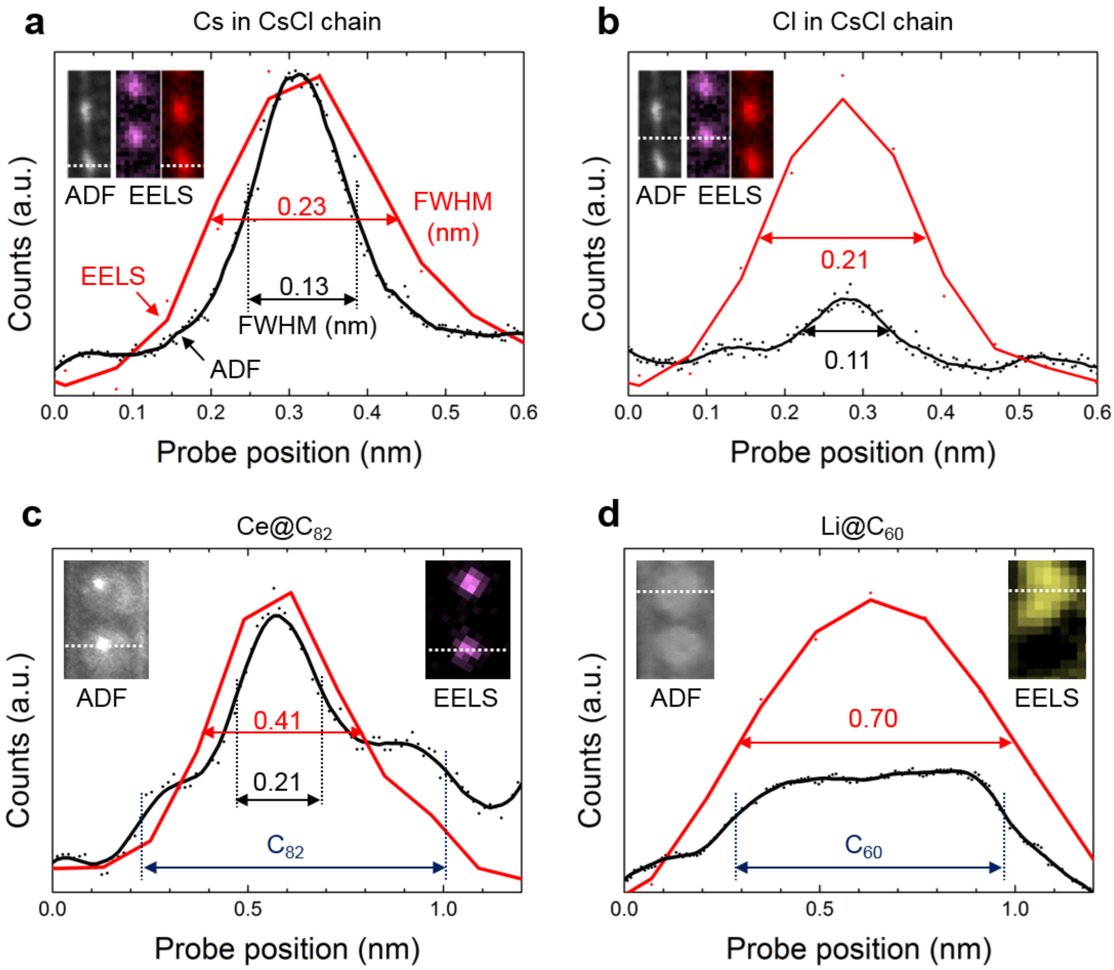

Supplementary Figure 7: Difference in profiles of ADF images and EELS signals for a single atom. (**a-d**) line profiles of ADF images (black line) and the corresponding EELS signals (red line) for Cs, Cl atoms in a CsCl chain, a Ce atom inside a C_{82}[1] and a Li atom inside a C_{60}, respectively. Line profiles are taken along the white lines in the insets. In the case of Cs, Cl and Ce atoms, the EELS profiles are broadened than ADF profiles. The difference is attributed to the EELS delocalization. Although there is no visible ADF contrast for the Li atom inside the C_{60} molecule, the fact that the EELS profile is wider than ADF profile of the C_{60} molecule reflects the contribution of the EELS delocalization for the Li atom.

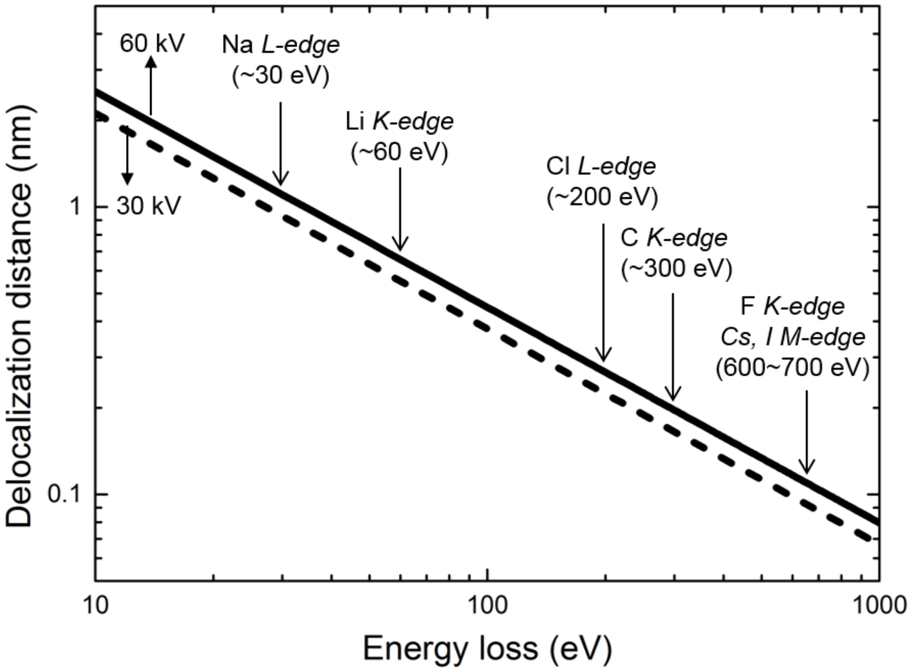

Supplementary Figure 8: Estimated localization distance corresponding to the energy loss. The broken and solid lines show the cases for the accelerating voltage of 30 and 60 kV, respectively. The vertical and horizontal axes are drawn in a logarithmic scale.

Supplementary note 1. Ionic atomic chains including “invisible” atoms

Supplementary Figures 1a–d present STEM annular dark-field (ADF) images of NaI, CsF, CsCl, and CsI atomic chains inside DWNTs. Although the ADF contrast profiles along the chains (Supplementary Fig. 1e) do not produce clear peaks for Na, F, and Cl atoms, there is sufficient space for those light atoms between two neighbouring Cs or I atoms, considering the atomic distance of CsI atomic chains. The average distance between two neighbouring Cs or I atoms is ordered according to CsF < NaI < CsCl < CsI, which simply reflects the sums of ionic radii for cation (Na^+ (102 pm), Cs^+ (170 pm)) and anion (F^- (133 pm), Cl^- (181 pm), and I^- (220 pm)). However, in the all cases presented here, the length between two neighbouring Cs or I atoms are shorter than twice the sums of ionic radii for cation and anion in bulk crystals. The fact suggests the shorter bond length of the ionic atomic chains than the bulk crystals.

Supplementary note 2. Single-atom spectroscopy of fluorine

Supplementary Figure 2 shows an atomic chain of CsF in a DWNT. Similar to the other cases (NaI and CsCl), the CsF atomic chain is supposed to be alternatingly aligned inside of the DWNT (Supplementary Fig. 2a). Supplementary Figure 2b–c shows the ADF image of CsF and corresponding elemental maps of Cs and F, based on Cs *M*-edge and F *K*-edge spectra. From those maps, it is clear that the F atoms are located exactly in the middle of two Cs atoms. However, the F map is not sophisticated to the extent like the Cl map, as shown in Fig. 3 of the main text. While some pixels have strong signals, others, especially those below pixels with high F *K*-edge intensity have weak or almost no signal. This means that fluctuations of the F atoms are induced by the electron irradiation and, finally, they are kicked out even from such a highly confined space during scanning.

The fine structure of the F *K*-edge is also affected by the coordination number, like the Li *K*-edge, as shown in Fig. 5 of the main text. The main peak of the F *K*-edge of CsF atomic chains is located at lower energy than in case of the 2 × 2 structure, as shown in Supplementary Fig. 2e. The electron affinity of the F atoms in CsF atomic chains should be higher than in the 2 × 2 structure because of the weaker Coulomb repulsion among nearest neighbouring F atoms. Thus, the lower binding energy of the F 1s electrons in CsF atomic chains causes the red shift in the F *K*-edge.

Supplementary note 3. Confidence level of invisible-atom detection

To consider the accuracy of our measurements, we estimated the signal–to-noise ratio (SNR) of our experimental data. We adopted the following expression for SNR in EELS, developed by Egerton[2].

$$\mathrm{SNR} = I_k/(I_k + hI_b)^{1/2} \qquad (1)$$

I_k and I_b are integration regions of the core-loss signal and the background (Supplementary Fig. 4a), respectively. h is a dimensionless parameter defined as $h = [I_b + var(I_b)]/I_b$ and represents the error caused by the background extrapolation. For our experiments, SNR for the Li *K*-edge corrected for even a single atom (Supplementary Fig. 4a) can be estimated to be approximately 8 (*h* = 10) by choosing the background fitting and integration region (Γ and Δ in Supplementary Fig. 4a,

respectively) carefully to avoid a large value of h.

In addition, the obtained signal of the Li K-edge is located sufficiently above the noise level. Supplementary Figure 4c shows the colour variation of the Li elemental map as a function of the standard variation of the signal noise σ, measured from the vacuum area far from the sample. In this map, the zigzag area has the intensity of more than 4σ. These values demonstrate a high confidence level of our results.

Supplementary note 4. Estimation of the delocalization distance

The broadening of EELS profile can be expressed by a distance between the incident beam and the target atom, called the impact parameter b. In the classical theory, the delocalization distance L is defined as the maximum value of b in which the dynamic screening does not take place and roughly determined by the velocity of incident electron v and the angular frequency of an atomic electron ω; $L = v/\omega$[3]. If we simply assume the absorption energy to excite the atomic electron as $\Delta E = (h/2\pi)\omega$, L is inversely proportional to the absorption energy. In addition, by introducing the following relation $\gamma m_0 v = h/\lambda$ (de Broglie relation) in which λ, γm_0, and h denote the wave length, effective electron mass, and Plank's constant, respectively, L is written as

$$L \approx \frac{v}{\omega} = \frac{h}{\gamma m_0 \lambda} \cdot \frac{h}{2\pi E} = \frac{\lambda}{2\pi\theta_E}, \qquad (2)$$

where θ_E is the characteristic scattering angle defined by $\theta_E = E/\gamma m_0 v^2$. For practical usage, L_{50} in which 50% of the inelastically scattered electrons are contained, is commonly used to estimate the degree of delocalization. L_{50} can be derived by adding cutoff functions to L, which is then approximated by

$$L_{50} \approx \frac{0.44\lambda}{\theta_E{}^{3/4}}. \qquad (3)$$

Supplementary Figure 8 presents the L_{50} values as function of the energy loss at the accelerating voltages of 30 (broken line) and 60 (solid line) kV. Much more detailed discussion has been reported by Egerton, Muller and Silcox[3–5], for example.

Supplementary note 5. Origin of localized EELS detectable area in 1D ionic crystals.

In Table 1 in main text, the experimentally measured EELS detectable distance for Li or Na atoms in 1D ionic crystals is much smaller than one for a Li atom inside a C_{60} molecule, even though the theoretically estimated EELS delocalization are larger due to the different acceleration voltages used (60 kV instead of 30kV). One of the reason for this discrepancy can be simply explained by the difference in the atomic motion. Light elements in 1D ionic crystal should be more confined (0.4nm) because of the robust ionic interaction between neighboring counter ions, while a Li atom inside C_{60} molecule can move rather freely in a larger space (0.7nm). A strong screening effect of the heavier atoms aside in 1D crystal may also contribute to reduce the delocalization effect of low-lying EELS edge.

Supplementary references

1. Suenaga, K., Iizumi, Y. & Okazaki, T. Single atom spectroscopy with reduced delocalization effect using a 30 kV-STEM. *Eur. Phys. J. Appl. Phys.* **54,** 33508 (2011).

2. Egerton, R. A revised expression for signal/noise ratio in EELS. *Ultramicroscopy* **9,** 387–390 (1982).

3. Egerton, R. F. Limits to the spatial, energy and momentum resolution of electron energy-loss spectroscopy. *Ultramicroscopy* **107,** 575–586 (2007).

4. Egerton, R. F. *Electron energy-loss spectroscopy in the electron microscope Third Edition*. (Springer, 2011).

5. Muller, D. & Silcox, J. Delocalization in inelastic scattering. *Ultramicroscopy* **59,** 195–213 (1995).
